# The combination of olaparib and camptothecin for effective radiosensitization

**DOI:** 10.1186/1748-717X-7-62

**Published:** 2012-04-23

**Authors:** Katsutoshi Miura, Koh-ichi Sakata, Masanori Someya, Yoshihisa Matsumoto, Hideki Matsumoto, Akihisa Takahashi, Masato Hareyama

**Affiliations:** 1 Department of Radiology, Sapporo Medical University, School of Medicine, S1W16, Chuo-Ku, Sapporo, 060-8543, Japan; 2 Tokyo Institute of Technology, Research Laboratory for Nuclear Reactors, N1-30 2-12-1 Ookayama, Meguro-ku, Tokyo, 152-8550, Japan; 3 Department of Oncology, Biomedical Imaging Research Center, University of Fukui, Eiheiji-cho, Fukui, 910-1193, Japan; 4 Advanced Scientific Research Leaders Development Unit, Gunma University, 3-39-22 Showa-machi, Maebashi, Gunma, 371-8511, Japan

**Keywords:** PARP inihibitor, Olaparib, Camptothecin, Topoisomerase I inhibitor, Radiosensitization

## Abstract

**Background:**

Poly (ADP-ribose) polymerase-1 (PARP-1) is a key enzyme involved in the repair of radiation-induced single-strand DNA breaks. PARP inhibitors such as olaparib (KU-0059436, AZD-2281) enhance tumor sensitivity to radiation and to topoisomerase I inhibitors like camptothecin (CPT). Olaparib is an orally bioavailable inhibitor of PARP-1 and PARP-2 that has been tested in multiple clinical trials. The purpose of this study was to investigate the characteristics of the sensitizing effect of olaparib for radiation and CPT in order to support clinical application of this agent.

**Methods:**

DLD-1 cells (a human colorectal cancer cell line) and H1299 cells (a non-small cell lung cancer cell line) with differences of p53 gene status were used. The survival of these cells was determined by clonogenic assay after treatment with drugs and X-ray irradiation. The γH2AX focus formation assay was performed to examine the influence of olaparib on induction and repair of double-stranded DNA breaks after exposure to radiation or CPT.

**Results:**

A radiosensitizing effect of olaparib was seen even at 0.01 μM. Its radiosensitizing effect after exposure for 2 h was similar to that after 24 h. H1299 cells with depletion or mutation of p53 were more radioresistant than H1299 cells with wild-type p53. However, similar enhancement of radiosensitization by olaparib was observed with all of the tested cell lines regardless of the p53 status. Olaparib also sensitized cells to CPT. This sensitizing effect was seen at low concentrations of olaparib such as 0.01 μM, and its sensitizing effect was the same at both 0.01 μM and 1 μM. The combination of olaparib and CPT had a stronger radiosensitizing effect. The results of the γH2AX focus assay corresponded with the clonogenic assay findings.

**Conclusion:**

Olaparib enhanced sensitivity to radiation and CPT at low concentrations and after relatively short exposure times such as 2 h. The radiosensitizing effect of olaprib was not dependent on the p53 status of tumor cells. These characteristics could be advantageous for clinical radiotherapy since tumor cells may be exposed to low concentrations of olaparib and/or may have different levels of p53 mutation. The combination of olaparib and CPT had a stronger radiosensitizing effect, indicating that combining a PARP inihibitor with a topoiomerase I inhibitor could be promising for clinical radiosensitization.

## Background

DNA is the principal target for the biologic effects of radiation. For radiotherapy, this comprises single strand breaks (SSB) and double-strand breaks (DSB) [1]. SSB are not directly cytotoxic but during DNA replication may generate potentially lethal DSB by collapse of stalled replication forks [2]. Radiation-induced SSB are primarily repaired by base excision repair [3], of which poly (ADP-ribose) polymerase-1 (PARP-1) is a key component. PARP-1 binds to SSB, activating poly ADP-ribosylation of itself and other proteins, triggering recruitment of repair factors and release of PARP-1 from the damaged site [4]. PARP inhibitors inhibit SSB repair and the unrepaired SSB generate collapsed replication forks which give rise to potentially lethal DSB, leading to radiosensitization [5]. Topoisomerase I poisons such as camptothecin (CPT) exert their cytotoxic effects by binding to and stabilizing the DNA helicase enzyme topoisomerase I. This enzyme plays a vital role in facilitating unwinding of the DNA double helix during DNA replication to relieve torsional strain. The mechanism involves temporary insertion of DNA breaks to allow unwinding, and an intermediate DNA/protein structure termed the ‘cleavable complex’ is generated. Topoisomerase I poisons stabilize the cleavable complex and extend the lifetime of the associated DNA strand breaks. PARP-1 is involved in the resolution of these DNA breaks, so inhibition of PARP activity increases the yield of unrepaired DNA damage and consequent cell death [6].

Olaparib (KU-0059436, AZD-2281) is an orally bioavailable inhibitor of PARP-1 and PARP-2 and a phase 1 trial using olaparib has been reported [7]. The purpose of this study was to examine characteristics of sensitizing effects of olaparib to radiation and a topoisomerase I inhibitor, CPT for clinical application of olaparib.

We found that olaparib sensitized cells to radiation and also CPT, even at low concentrations such as 0.01 μM. The radiosensitizing effect of olaparib at exposure for 2 h was similar as for 24 h. These characteristics can be advantageous in radiotherapy since tumor cells may have low concentrations of olaparib due to the limited ability of drugs to penetrate tumor tissue. We also examined the radiosensitizing effects of the combined use of olaparib and CPT.

## Methods

### [Drugs, cells and cell culture]

Olaparib was obtained from JS Research Chemicals Trading (Germany). CPT was obtained from Sigma Chemical Company. DLD-1 is a human colorectal carcinoma cell line. H1299 is a *p53*-null non-small lung carcinoma cell line. H1299 cells were transfected with pCMV-Bam-Neo vector (neo) alone (as a control) and with wild-type p53 (wt) or mutated p53 gene [8,9]. H1299/m*p143* (m143)*,* H1299/m*p175* (m175)*,* and H1299/m*p248* (m248) were made with transfection of mutated p53 gene into codon 143, 175, 248 of H1299 cells, respectively. To determine whether *p53* mRNA and the protein were stably expressed in these transfectants, we analyzed the reverse transcriptase-polymerase chain reaction restriction fragment length polymorphism on the sequence of transfected codon of the *p53* gene for the mRNA. We also performed Western blot analysis for the protein. H1299 (wt, m248, m175, m143, neo) cells were cultured in MEM medium supplemented with 10% fetal calf serum. DLD-1 was cultured in RPMI-1640 medium supplemented with 10% fetal calf serum. All these cell lines were cultured at 37°C.

### [Cell culture and clonogenic assay]

The survival of these cells after various treatments such as drug treatment and X-ray irradiation was determined as their colony-forming ability. The time between plating cells and treatments such as radiation and/or drug exposure was 24 h. Experiments were repeated three times. Mean values and standard error of the mean were expressed. We tested the significance at the *p* < 0.05 for our observations. The X-ray dose-survival curves were fitted to the linear quadratic equation, survival fraction = exp (−αD-βD^2^), where D is the X-ray dose. D_37_, the X-ray dose giving 37% survival was calculated from the fitted linear quadratic equation. Enhancement ratio was calculated as the D_37_ of untreated cells divided by that of treated cells. Since CPT have the cell killing effects, we excluded their cell killing effects in the calculation of enhancement ratio. We made the hypothetical survival curve in which the value of survival fraction was 1 at 0 Gy in the combined treatment of CPT and radiation.

### [γH2AX focus assay]

Mouse monoclonal anti-γH2AX (Upstate, NY, USA) antibody and rabbit polyclonal anti- Rad51 (Oncogene, CA, USA) antibody were used. DLD-1 cells were exposed to olaparib (1 μM) for 8 h and/or CPT (0.01 μM) for 6 h. Since CPT has the cell killing effect, we used the shorter exposure time in CPT than olaparib. When cells were irradiated, 4 Gy of radiation was performed after 4 h exposure of olaparib and/or 2 h exposure of CPT. Then, cells were applied on slide glass at 24 h after irradiation. Cells diluted to appropriate numbers were grown on a glass slide and fixed with cold methanol for 20 min, rinsed with cold acetone for 10 seconds, and then air-dried. Above mentioned antibodies to γH2AX or Rad51 were used as the primary antibody. Alexa-546-conjugated anti-mouse IgG (Molecular Probes) were used for visualization of γH2AX and Rad51. Slides were mounted with antifade reagent (Mounting medium, DAKO). Foci were observed with an Olympus fluorescent microscope under 10 × 100 oil immersion. For quantification of foci, clear and easily distinguished dots of certain brightness were counted as positive foci. The number of foci was counted in 100 cells of sample each time point by visual inspection and average number of foci per cell was calculated. Cells that had 5 or more of Rad51 were considered as Rad51 positive.

### [Flowcytometry]

For estimation of the distribution of cells in different phases of the cell cycle, FACS-analysis of cells stained with propidium iodide (PI) was performed. Harvested cells were fixed in 70% of ethanol and stored at 4°C until analysis. Fixed cells were washed once in PBS, incubated with PBS supplemented with RNase A (0.25 mg/ml) for 30 min at 37°C and stained in a PI-containing solution (50 μg PI/ml in PBS) for 10 min at 4°C. The cells were analyzed on a flowcytometer (FACS Calibur, Becton Dickinson, USA).

## Results

### [Radiosensitizing effect of olaparib at various exposure times and concentrations]

**Figure 1:**
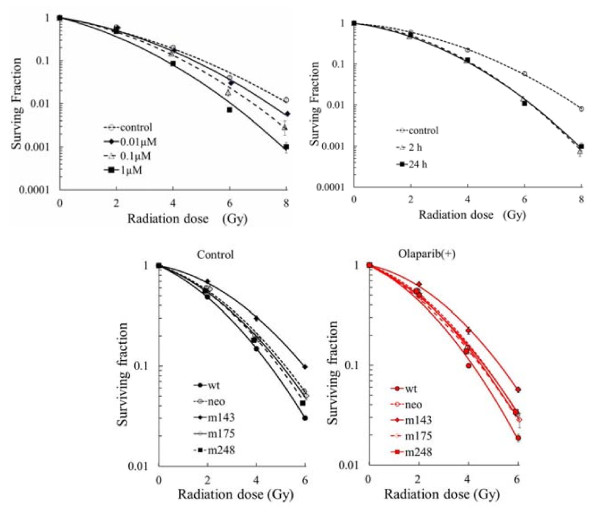
**a**. **The relationship between concentrations of olaparib and radiosensitizing effects.** DLD-1 cells were treated with various concentrations of olaparib for 1 h before radiation and 24 h after radiation. **b**. **The relationship between exposure time (1 h or 24 h) of olaparib and radiosensitizing effects.** DLD-1 cells were treated with 1 μM of olaparib for 1 h before radiation and 1 h or 23 h after radiation. **c**. **Clonogenic cell survival assay of H1299 cells with various status of p53 gene.** m*143,* m*175,* and m*248* were made with transfection of mutated p53 gene into codon 143, 175, 248 of H1299 cells, respectively. “Control”: treated with radiation alone; “Olaparib (+)”:treated with radiation and olaparib. Cells were treated with 1 μM of olaparib for 1 h before radiation and 2 h after radiation.

Figure 1a demonstrates the relationship between the concentration of olaparib and its radiosensitizing effect. The surviving fraction was significantly reduced after exposure to 0.01 μM olaparib (*p* < 0.05) compared with the control (radiation alone), indicating that this agent had a radiosensitizing effect even at low concentrations. The radiosensitizing effect increased in a concentration-dependent manner. Figure 1b demonstrates the relationship between the exposure time and the radiosensitizing effect of olaparib. A radiosensitizing effect was seen after only 2 h of exposure, and it was similar to the effect after 24 h of exposure.

Additional file 1 shows the relationship between the olaparib concentration and its radiosensitizing effect on DLD-1 cells. These cells were treated with 4 Gy of radiation and were exposed to various concentrations of olaparib for 1 h before irradiation and 24 h after irradiation. Additional file 2 shows the relationship between the olaparib exposure time and its radiosensitizing effect on DLD-1 cells. The results displayed in Additional file 1 and Additional file 2 correspond with those shown in Figure 1a and 1b. Cells were treated with 4 Gy of radiation and were exposed to 1 μM olaparib for 1 h before irradiation and various times after irradiation. In these experiments, a radiation dose of 4 Gy was used since it killed more cells than 2 Gy, and the difference between various treatments was clearer at 4 Gy.

Olaparib did not decrease plating efficiency at any of the exposure times or concentrations examined in this study.

### [Radiosensitizing effect of olaparib on human cancer cells with different p53 statuses]

We examined whether the p53 status of tumor cells influenced the enhancement of radiosensitivity by olaparib. To assess the influence of p53 status, we used five human lung cancer cell lines with identical genotypes except for p53 (wt, m143*,* m175*,* m248*,* and neo) and found that wt cells were significantly more radiosensitive than m143 and neo cells (*p* < 0.01). Conversely, m143 cells were significantly more radioresistant than m248 and wt cells (*p* < 0.01). These results indicated that the p53 status influenced the radiosensitivity of H1299 cell lines.

Olaparib enhanced the radiosensitivity of all tumor cell lines examined, especially at higher radiation doses (Figure 1c). Table 1 shows the values of α and β in the linear quadratic equation for calculating the surviving fraction, i.e., surviving fraction = exp (−αD-βD^2^). Similar enhancement of radiosensitization was observed with all cell lines tested regardless of the p53 status. Exposure to olaparib alone (1 μM, 3 h) did not affect the plating efficiency of any cell line examined in this study.

**Table 1:** Radiation enhancement ratio by olaparib

		**α**		**β**		**enhancement ratio**
wt	Control	0.27	±0.082	0.053	±0.016	1.1
	Olaparib(+)	0.28	±0.022	0.065	±0.002	
neo	Control	0.19	±0.015	0.049	±0.002	1.1
	Olaparib(+)	0.22	±0.039	0.059	±0.008	
m143	Control	0.11	±0.030	0.047	±0.005	1.2
	Olaparib(+)	0.13	±0.052	0.060	±0.009	
m175	Control	0.22	±0.086	0.048	±0.015	1.2
	Olaparib(+)	0.29	±0.076	0.051	±0.012	
m248	Control	0.21	±0.094	0.054	±0.013	1.1
	Olaparib(+)	0.26	±0.027	0.053	±0.005	

### [Sensitization to CPT by olaparib]

**Figure 2:**
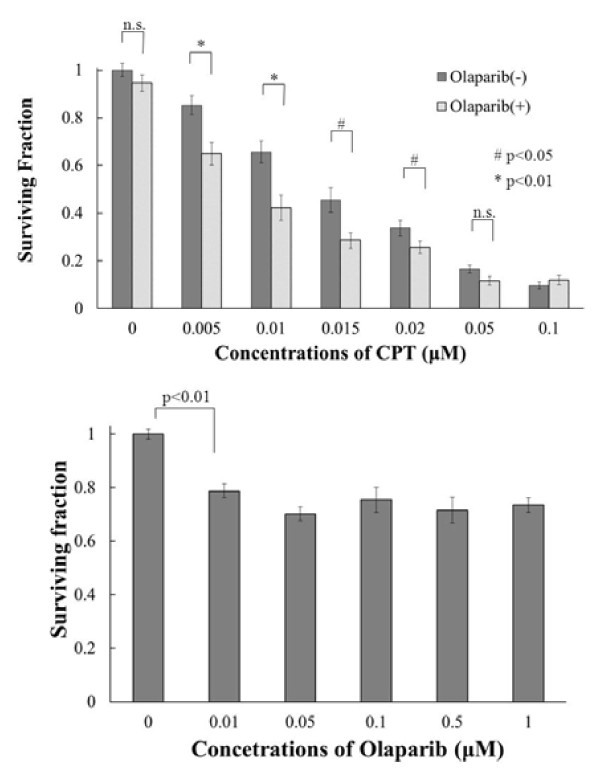
**a**. **The effect of olaparib on CPT toxicity.** DLD-1 cells were treated with 1 μM of olaparib and various cocentrations of CPT for 6 h. Values shown are the mean ± standard error of the mean. **b**. **The relationship between concentrations of olaparib and sensitizing effects to CPT in DLD-1 cells.** Cells were treated with 0.01 μM of CPT and various concentrations of olaparib for 6 h. Values shown are the mean ± standard error of the mean.

In order to examine the influence of olaparib on the sensitivity of DLD-1 cells to the topoisomerase I inhibitor CPT, we performed a clonogenic cell survival assay. Figure 2a demonstrates that olaparib sensitized the cells to CPT. This effect was seen even at low concentrations of CPT, such as 0.005 μM, and it was maximal around 0.01 or 0.015 μM.

We also examined the influence of the concentration of olaparib on its sensitizing effect, especially in regard to low concentrations (Figure 2b). A sensitizing effect was evident at only 0.01 μM and the strength of the sensitizing effect was the same at both 0.05 μM and 1 μM.

### [Radiosensitization with olaparib plus CPT]

**Figure 3:**
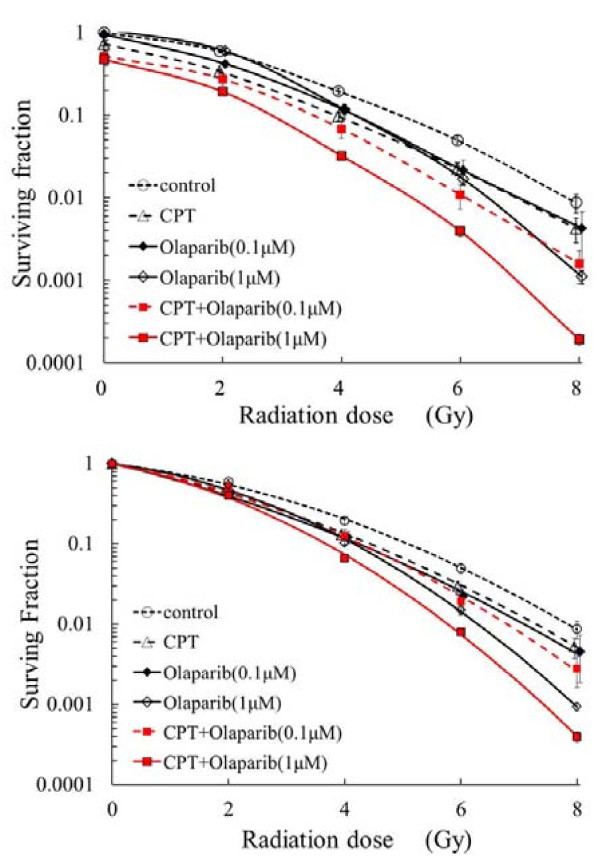
**a**. **Cell survivals with radiation combined with olaparib and/or CPT.** DLD-1 cells were treated with 0.1 or 1 μM of olaparib and/or 0.01 μM of CPT for 4 h before irradiation and for 2 h after radiation. **b**. **Radiosensitization with olaparib and/or CPT.** Figure 3b was made by modifying Figure 3a. The survival fraction of CPT at 0 Gy in Figure 3a was corrected to 1.

Figure 3a shows the surviving fraction of DLD-1 cells after irradiation combined with exposure to olaparib and/or CPT. Since CPT had a cytotoxic effect, the surviving fraction was less than 1 at 0 Gy. Compared with radiation alone, radiation combined with olaparib or CPT significantly reduced the surviving fraction of DLD-1 cells (*p* < 0.01). The greatest reduction of the surviving fraction was achieved by the combination of 1 μM of olaparib and CPT (*p* < 0.001).

As shown in Figure 3b, the surviving fraction of cells exposed to CPT at 0 Gy of irradiation has been adjusted to 1. It can be seen that the combination of olaparib and CPT had the strongest effect since olaparib increased sensitivity to both radiation and CPT. The enhancement ratio (D_37_) for 0.1 μM olaparib and CPT was 1.37, while that for 1 μM olaparib and CPT was 1.45. In addition, the enhancement ratio (D_37_) for 1 μM olaparib alone was 1.20 and that for CPT alone was 1.33.

### [γH2AX focus formation assay after irradiation or CPT]

**Figure 4:**
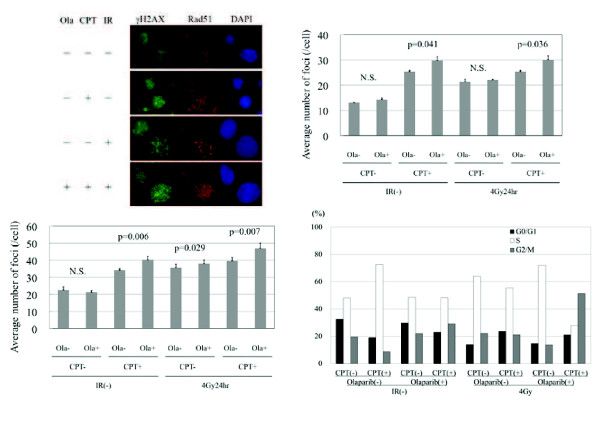
**γH2AX foci at 24 hours after irradiation and cell cycle distribution.** DLD-1 cells were treated with 4 Gy of radiation and/or 1 μM of olaparib for 8 h and/or 10nM of CPT for 6 h. (**a**) Foci of γH2AX and Rad51 in DLD-1 cells treated with various combinations of olaparib, CPT, or irradiation (IR). DAPI(4',6-diamidino-2-phenylindole) was used to stain nucleus. Ola(+): with olaparib. IR(−): without irradiation. (**b**) Results of cells that were positive for Rad51 foci. (**c**) Results of cells that were negative for Rad51 foci. (**d**) Cell cycle distributions.

In order to examine the relationship between olaparib-induced sensitization to radiation or CPT and the induction of double-stranded DNA breaks (DSB), we counted residual γH2AX foci at 24 h after irradiation and 20 h after treatment with olaparib and/or CPT (Figure 4). We also analyzed γH2AX foci in relation to the existence of Rad51 foci. Induction of Rad51 by irradiation is cell-cycle dependent, since it is preferentially formed in the S and G2/M phases [10]. Thus, the results of the γH2AX focus assay can be evaluated by taking into consideration the influence of the cell cycle.

Figure 4a shows the γH2AX and Rad51 foci in untreated DLD-1 cells, cells treated with irradiation (IR), and cells treated with CPT. Figure 4b presents the results for cells with Rad51 foci. There was no significant difference between the control cells (Ola-, CPT-, IR-) and cells treated with olaparib (Ola+, CPT-, IR-), suggesting that olaparib alone did not induce DSB. Cells treated with radiation (Ola-, CPT-, IR+) had significantly more foci than control cells (Ola-, CPT-, IR-) as did cells treated with CPT (Ola-, CPT+, IR-), indicating that radiation or CPT induced DSB. Cells exposed to radiation combined with olaparib (Ola+, CPT-, IR+) had significantly more foci than cells treated with radiation alone (Ola-, CPT-, IR+). In addition, cells exposed to CPT combined with olaparib (Ola+, CPT+, IR-) showed significantly more foci than cells treated with CPT alone (Ola-, CPT+, IR-), revealing that olaparib enhanced the induction of DSB by radiation or CPT. Cells exposed to radiation combined with olaparib (Ola+, CPT-, IR+) had a similar number of foci as cells exposed to radiation combined with CPT (Ola-, CPT+, IR+), showing that olaparib and CPT caused similar enhancement of the induction of DSB by radiation. Cells exposed to radiation combined with olaparib and CPT (Ola+, CPT+, IR+) had the most foci among all of the treatment groups.

Figure 4c presents the results for cells without Rad51 foci. The findings are similar to those shown in Figure 4b. However, the enhancement of radiation-induced DSB by olaparib and/or CPT was less marked in cells without Rad51 foci than in cells with Rad51 foci. There was no significant difference between radiation combined with olaparib (Ola+, CPT-, IR+) and radiation alone (Ola-, CPT-, IR+) in cells without Rad51 foci.

### [Effects of olaparib, CPT and radiation on the cell cycle]

Figure 4d shows the cell cycle after the same treatments as Figure 4b and c. There was no difference between the control cells (Ola-, CPT-, IR-) and cells treated with olaparib (Ola+, CPT-, IR-), indicating that olaparib alone did not change cell cycle distributions. Cells treated with radiation (Ola-, CPT-, IR+) or CPT (Ola-, CPT+, IR-) tended to have more S phase cells than the control cells (Ola-, CPT-, IR-). Radiation and olaparib (Ola+, CPT-, IR+) had the similar cell cycle distribution to radiation alone (Ola-, CPT-, IR+). In contrast, cells treated with CPT and olaparib (Ola+, CPT+, IR-) tended to have reduced S phase cells, compared with CPT alone (Ola-, CPT+, IR-). Cells treated with olaparib, CPT and radiation (Ola+, CPT+, IR+) had increased G2/M phase cells and reduced S phase cells, compared with the other treatments.

## Discussion

PARP inhibitors such as olaparib increase sensitivity to radiation and to topoisomerase I inhibitors such as CPT [4]. The concentration of olaparib tested in previous studies was 0.5 or 1 μM [5,11]. In a clinical study, the plasma concentration of olaparib reached 11.5 μM (5 μg/ml) after oral administration of 100 or 200 mg, which are doses that do not cause dose-limiting toxicity [7]. The present findings support the clinical usefulness of olaparib at oral doses of 100 to 200 mg. However, the ability of drugs to penetrate tumor tissue *in vivo* is often limited, so that some tumor cells may not be exposed to effective concentrations [12]. Therefore, it is possible that tumor cells may be exposed to much lower olaparib concentrations than 0.5 or 1 μM. For this reason, we examined the sensitizing effect of olaparib for radiation and CPT at very low concentrations.

Figure 1a demonstrates the relationship between the olaparib concentration and its radiosensitizing effect on DLD-1 cells. Although the radiosensitizing effect of olaparib showed a concentration-dependent increase, low concentrations (such as 0.01 μM) still had a radiosensitizing effect, indicating that effective radiosensitization achieved even for tumor cells exposed to low concentrations of this agent.

We also examined the relationship between the exposure time and the radiosensitizing effect of olaparib. Figure 1b demonstrates that the radiosensitizing effect of olaparib after exposure for 2 h was similar to that at 24 h, demonstrating that effective radiosensitization could be achieved even for tumor cells with a short exposure time. Olaparib enhances radiosensitivity by inhibiting the repair of SSB. Unrepaired SSB lead to collapse of replication forks that give rise to potentially lethal DSB, leading to radiosensitization [5]. Thus, SSB are converted to DSB during replication after exposure to olaparib [13]. Longer incubation times than 2 h after irradiation might be required to convert SSB to DSB since the cell cycle of DLD1 cells lasts for 24 h or longer. However, we found that 2 h of incubation with olaparib after irradiation is sufficient for radiosensitization and that exposure to this agent throughout the entire period of SSB repair is not necessary to enhance the generation of DSB.

In order to test the potential clinical value of olaparib, we also examined whether the p53 status of tumor cells influenced the enhancement of radiosensitization using five human lung cancer cell lines with identical genotypes except for p53 (wt, m143, m175, m248, and neo). We found that H1299 cells with depletion or mutation of p53 were more radioresistant than H1299 cells with wild-type p53, showing that p53 status influenced the radiosensitivity of these cell lines. There have been conflicting reports concerning the relationship between p53 status and radiosensitivity. Some authors have reported that p53 gene mutation decreases radiosensitivity [14], while others have reported an increase of radiosensitivity [15] or no correlation with radiosensitivity [16]. Because p53 regulates multiple aspects of the cell cycle, DNA repair, and apoptosis after irradiation [17], this protein not only determines cell death but also cell survival. Accordingly, cells with various p53 statuses could be more radiosensitive or radioresistant [18]. In the present study, the surviving fraction was reduced by exposure to olaparib for all of the cell lines tested with various p53 statuses. Enhancement of radiosensitization was similar for all of the cell lines regardless of p53 status (Table 1), indicating that the radiosensitizing effect of olaparib is independent of p53.

Next, we investigated the effect of olaparib on sensitivity to CPT. Figure 2a shows the relationship between the olaparib concentration and its CPT-sensitizing effect. Olaparib sensitized cells to the cytotoxic effect of CPT, even at a CPT concentration of only 0.005 μM. The combination of olaparib with CPT significantly reduced the surviving fraction over the range of CPT concentrations from 0.005 to 0.02 μM. We also examined the relationship between the sensitizing effect of olaparib and its concentration, especially at low concentrations (Figure 2b). A sensitizing effect was still apparent at a concentration of 0.01 μM and the effect seen at this level was similar to that obtained at 1 μM.

We then investigated the combined effects of olaparib, CPT, and radiation. Figure 3a displays the surviving fraction of DLD-1 cells after treatment with olaparib and/or CPT. The combination of radiation with olaparib or CPT significantly increased the lethal effect of radiation (*p* < 0.01), except when olaparib was combined with 2 Gy. The cytotoxic effect of olaparib, CPT, and radiation all together was much greater than that of radiation alone.

In order to compare the radiosensitizing effect of CPT with that of olaparib, we adjusted the surviving fraction at 0 Gy to 1 in Figure 3b, since CPT itself had a cytotoxic effect. Then we assessed the radiosensitivity of DLD-1 cells after treatment with olaparib or CPT. The radiosensitizing effect of 1 μM olaparib was similar to that of CPT at 4 Gy and greater than that of CPT at 6 and 8 Gy (*p* < 0.01). Since olaparib increased sensitivity to both radiation and CPT, the combination of olaparib and CPT had the strongest radiosensitizing effect. The enhancement ratio (D_37_) of olaparib plus CPT was larger than that for either olaparib alone or CPT alone. Thus, combination therapy with PARP inhibitors and topoisomerase I inhibitors may be a promising strategy for enhancing the effects of radiotherapy.

After DSB are induced by irradiation or cytotoxic drugs, H2AX is rapidly phosphorylated and there is always a constant number or percentage of γH2AX formed per DSB. Therefore, γH2AX is a sensitive and early indicator of DSB *in vitro* and *in vivo*[19]. Furthermore, residual γH2AX at 24 h after irradiation can be used to assess the radiosensitivity of cells or their ability to recover from damage and the efficiency of the cellular repair process [20]. Accordingly, we investigated residual γH2AX foci after exposure of cells to radiation, olaparib, and CPT. Olaparib alone did not increase γH2AX foci, while radiation or CPT did so. Radiation combined with olaparib and CPT produced the most foci among all of the treatment groups. These findings agreed with the results of clonogenic assays, suggesting that the sensitizing effect of olaparib for radiation or CPT is related to induction of DSB or to inhibiting the repair of DSB. We also used radiation-induced Rad51 foci to investigate the cell cycle dependence of γH2AX focus formation after various treatments. We found that enhancement of radiation-induced DSB by olaparib and CPT was greater in cells with Rad51 foci than in cells without Rad51 foci, indicating that olaparib had a stronger effect on the induction of DSB by radiation or CPT in the S and G2/M phases than in the G1 phase.

Figure 4d shows the cell cycle after the treatments with olaparib, CPT, or radiation.

Olaparib alone did not change cell cycle distributions. Cells treated with radiation or CPT had more S phase cells than the control cells, corresponding to that radiation [21] or CPT [22] arrests or delays cell cycle progression during the S phase. Cells treated with CPT and olaparib had less S phase cells than CPT alone. This might suggest that olaparib enhances the S phase specific cell killing effect of CPT [23]. The combination of olaparib, CPT and radiation reduced S phase and increased G2/M phase. This effect might result from S phase specific cell killing with CPT and G2/M arrest with radiation.

## Conclusion

Olaparib enhanced the sensitivity of radiation and CPT, even at low concentrations, such as 0.01 μM. The radiosensitizing effects of olaparib at exposure for 2 h had the similar as for 24 h. The radiosensitizing effect of olaprib did not depend on p53 status. These characteristics can be advantageous in clinical radiotherapy since tumor cells may have low concentrations of olaparib and/or p53 mutation. The combination of olaparib and CPT had the good radiosensitizing effects, indicating that the combination of PARP inhibitor and topoiomerase I inhibitor can be promising as a method of clinical radiosensitization.

## Competing interest

The authors declare that they have no competing interests.

## Authors’ contributions

KM performed experiments and drafted the manuscript. KS conceived of the study and drafted the manuscript. MS performed γ-H2AX assay. YM participated importantly in the conception of the study and helped to draft the manuscript. HM and AT made H1299 cells with various p53 status and analyzed these characteristics of cells. MH participated importantly in the conception and design and helped to draft the manuscript. All authors read and approved the final manuscript.

## Supplementary Material

Additional file 1 **The relationship between concentrations of olaparib and radiosensitizing effects.** DLD-1 cells were treated with 4 Gy of radiation and various concentrations of olaparib for 1 h before radiation and 24 h after radiation. Click here for file

Additional file 2 **The relationship between exposure time of olaparib and radiosensitizing effects.** DLD-1 cells were treated with 4 Gy of radiation and 1 μM of olaparib for 1 h before radiation and various times after radiation. Click here for file

## References

[B1] Hall EJ Hall EJ, Giaccia AM DNA strand breaks and chromosomal aberrations Radiobiology for the radiologist, ed 2006 6 Lippincott Williams & Wilkins, Philadelphia 16 29

[B2] Saleh-Gohari N Bryant HE Schultz N Parker KM Cassel T Helleday T Spontaneous homologous recombination is induced by collapsed replication forks that are caused by ebdogenous DNA single-strand breaks Mol Cell Biol 2005 25 16 7158 7169 10.1128/MCB.25.16.7158-7169.2005 16055725 PMC1190269

[B3] Caldecott KW Protein-protein inetractions during mammalian DNA single-strand break repair Biochem Soc Trans 2003 31 247 251 12546695 10.1042/bst0310247

[B4] Chalmers AJ The potential role and application of PARP inhibitors in cancer treatment Brit Med Bull 2009 89 23 40 19208614 10.1093/bmb/ldp005

[B5] Dungey FA Loser DA Chalmers AJ Replication-dependent radiosensitization of human glioma cells by inhibition of poly (ADP-ribose) polymerase: mechanisms and therapeutic potential Int J Radiat Oncol Biol Phys 2008 72 4 1188 1197 10.1016/j.ijrobp.2008.07.031 18954712

[B6] Smith LM Willmore E Austin CA Curtin NJ The novel poly (ADP-ribose) polymerase inhibitor, AG14361, sensitizes celss to topoisomerase I poisons by increasing the persistence of DNA strand breaks Clin Cancer Res 2005 11 23 8449 8457 10.1158/1078-0432.CCR-05-1224 16322308

[B7] Fong PC Boss DS Timothy A Tutt A Wu P Mergui-Roelvink M Mortimer P Swaisland H Lau A O’Connor MJ Ashworth A Carmichael J Kaye SB Schellens JHM de Bono JS inhibition of poly (ADP-ribose) polymerase in tumors from BRCA mutation carriers N Engl J Med 2009 361 2 123 134 10.1056/NEJMoa0900212 19553641

[B8] Jin Z-H Matsumoto H Hayashi S Hatashita M Ohtsubo T Shioura H Kitai R Kano E p53-independent thermosensitization by mitomycin c in human non-small-cell cancer cells Int J Radiat Oncol Biol Phys 2004 59 852 860 10.1016/j.ijrobp.2004.01.033 15183489

[B9] Kawashima D Soga M Takeuchi R Matsumoto H Ohtsuka K Molecular caperone inducers facilitate the functional restoration of temperature-sensitive mutant p53 protein Thermal Med 2010 26 1 17 10.3191/thermalmed.26.1

[B10] Yuan SF Chang H Lee EY Ionizing radiation-induced Rad51 nuclear focus formation is cell cycle-regulated and defective in both ATM-/- and c-Abl-/- cells Mutat Res 2003 525 85 92 10.1016/S0027-5107(03)00009-5 12650908

[B11] Loser DA Shibata A Shibata AK Woodbine LJ Jeggo PA Chalmers AJ Sensitization to radiation and alkylating agents by inhibitors of poly (ADP-ribose) polymerase is enhanced incells deficient in DNA double-strand break repair Mol Cacncer ther 2010 9 6 1775 1787 10.1158/1535-7163.MCT-09-1027 PMC2884153 20530711

[B12] Tredan O Galmarini CM Patel K Tannock IF Drug resistamce and the solid tumor microenvironment J Natl Cancer Inst 2007 99 1441 1454 10.1093/jnci/djm135 17895480

[B13] Bryant HE Schultz N Thomas HD Parker KM Flower D Lopez E Kyle S Meuth M Curtin NJ Helleday T Specific killing of BRCA2-deficient tumours with inhibitors of poly (ADP-ribose) polymerase Nature 2005 434 913 917 10.1038/nature03443 15829966

[B14] Sinclair WK The combined effects of hydroxyurea and x-rays on Chinese hamster cells in vitro Cancer Res 1968 28 198 206 5641513

[B15] Brachman DG Beckett M Graves D Haraf D Vokes E Weichselbaum RR p53 mutation does not correlate with radiosensitivity in 24 head and neck cancer cell lines Cancer Res 1993 53 16 3667 3669 8339273

[B16] Slichenmyer WJ Nelson WG Slebos RJ Kastan MB Loss of a p53-associated G1 checkpoint does not decrease cell survival following DNA damage Cancer Res 1993 53 18 4164 4168 8364909

[B17] Hall EJ Hall EJ, Giaccia AM Repair of radiation damage and the dose-rate effect Radiobiology for the radiologist, ed 2006 6 Lippincott Williams & Wilkins, Philadelphia 60 84

[B18] Takahashi A Matsumoto H Yuki K Yasumoto J Kajiwara A Aoki M Furusawa Y Ohnishi K Ohnishi T High-LET radiation enhanced apoptosis but not necrosis regardless of p53 status Int J Radiat Oncol Biol Phys 2004 60 2 591 597 15380596 10.1016/j.ijrobp.2004.05.062

[B19] Rogakou EP Nieves-Neira W Boon C Pommier Y Bonner WM Initiation of DNA fragmentation during apoptosis induces phosphorylation of H2AX histone at serine 139 J Biol Chem 2000 275 9390 9395 10.1074/jbc.275.13.9390 10734083

[B20] Klokov D MacPhail SM Banath JP Byrne JP Olove PL Phosphorylated histone H2AX in trlation to cell survival in tumor cells and xenografts exposed to single and fractionated doses of X-rays Radiother Oncol 2006 80 223 229 10.1016/j.radonc.2006.07.026 16905207

[B21] Guo CY D’Anna JA Larner JM The radiation-induced S-phase checkpoint is independent of CDKN1A Radiat Res 1999 151 125 132 10.2307/3579762 9952296

[B22] Shao R-G Cao C-X Shimizu T O’Connor PM Kohn KW Pommier Y Abrogation of an S-phase checkpoint and potentiation of camptothecin cytotoxicity by 7-hydroxystaurosporine (UCN-01) in human cancer cell lines, possibly influenced by p53 function Cancer Res 1997 57 4029 4035 9307289

[B23] Hsiang Y-H Lihou MG Liu LF Arrest of replication forks by drug-stabilized topoisomerase I-DNA cleavable complexes as a mechanism of cell killing by camptothecin Cancer Res 1989 49 5077 5082 2548710

