# Supplementary figures and images for: The combination of olaparib and camptothecin for effective radiosensitization
Source: Radiat Oncol. 2012 Apr 23;7:62. doi: 10.1186/1748-717X-7-62 (PMC3430568; doi:10.1186/1748-717X-7-62)

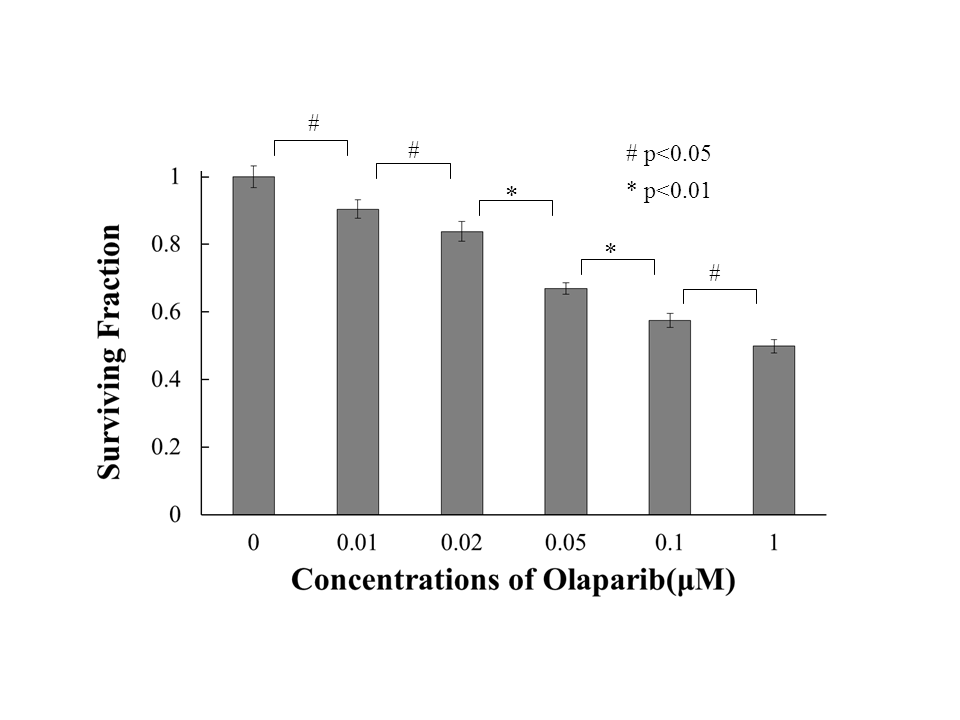

Supplement: Additional file 1 — The relationship between concentrations of olaparib and radiosensitizing effects. DLD-1 cells were treated with 4 Gy of radiation and various concentrations of olaparib for 1 h before radiation and 24 h after radiation. [file 1748-717X-7-62-S1.tiff]

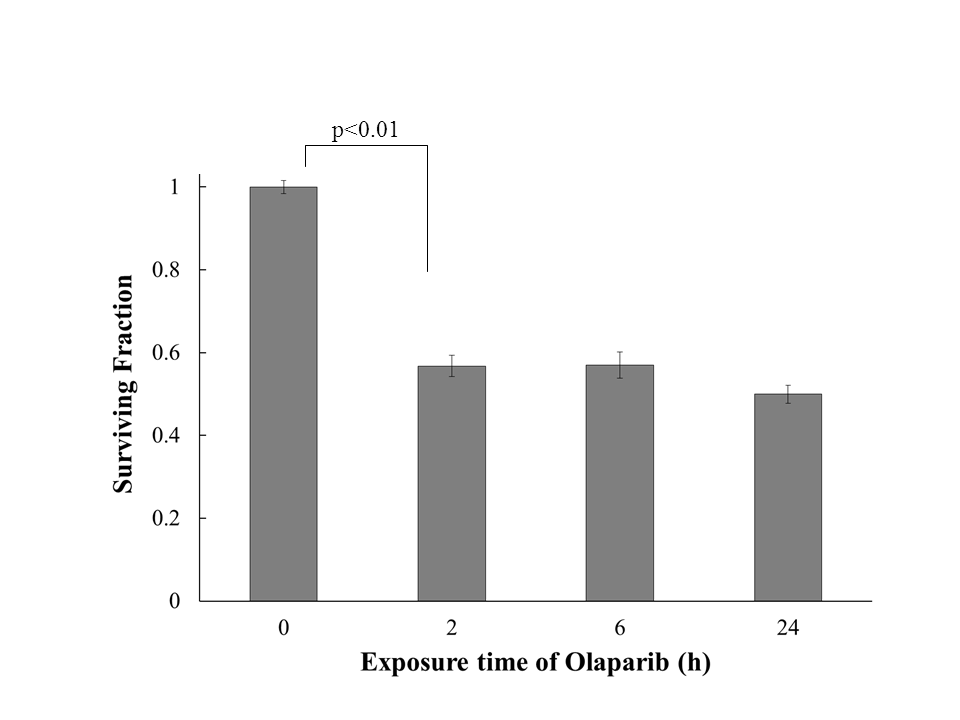

Supplement: Additional file 2 — The relationship between exposure time of olaparib and radiosensitizing effects. DLD-1 cells were treated with 4 Gy of radiation and 1 μM of olaparib for 1 h before radiation and various times after radiation. [file 1748-717X-7-62-S2.tiff]
